# Creating Stories Using TimeSlips With People Living With Dementia

**DOI:** 10.1093/geroni/igaa057.2421

**Published:** 2020-12-16

**Authors:** Emily Ihara, Kendall Barrett, Catherine Tompkins, Megumi Inoue, Kari Hanson

**Affiliations:** 1 George Mason University, Fairfax, Virginia, United States; 2 TimeSlips, Milwaukee, Wisconsin, United States

## Abstract

TimeSlips is an award-winning, non-pharmacologic, creative storytelling intervention designed specifically for individuals living with dementia by Anne Basting. As part of the NextGen pilot program, George Mason University implemented TimeSlips at a dementia-specific adult day health care center with undergraduate and graduate students trained as facilitators. They worked with two different populations living with ADRD – an early-stage group and a moderate- to late-stage group. Students who participated reflected upon their experiences and noted that they learned so much about the importance of open-ended questions and accepting even one-word answers. One student stated: “This makes so much sense, as I have noticed participants take these open-ended questions and deliver beautifully unexpected and clever responses.” The implementation was different for these two groups, and our analysis of the content and process indicate that consistency and person-centeredness are key to participants’ expression of pride and joy in their accomplishments.

